# The Influence of Acute Sprint Interval Training on the Cognitive Performance of Male Basketball Players: An Investigation of Expertise-Related Differences

**DOI:** 10.3390/ijerph20064719

**Published:** 2023-03-07

**Authors:** Egemen Mancı, Fabian Herold, Erkan Günay, Çağdaş Güdücü, Notger G. Müller, Cem Ş. Bediz

**Affiliations:** 1 Faculty of Sport Science, Dokuz Eylül University, Izmir 35320, Turkey; 2 Research Group Degenerative and Chronic Diseases, Movement, Faculty of Health Sciences Brandenburg, University of Potsdam, 14476 Potsdam, Germany; 3 Faculty of Sport Science, Celal Bayar University, Manisa 45140, Turkey; 4 Department of Biophysics, Dokuz Eylül University, Izmir 35320, Turkey; 5 Department of Physiology, University of Kyrenia, Kyrenia 99138, Cyprus

**Keywords:** sport psychology, choice reaction time, neuropsychological testing, sport/athletic technique, factors in sport performance

## Abstract

Highly developed cognitive abilities are an important prerequisite for reaching elite athletic levels. This study aimed to investigate the effect of an acute sprint interval training (SIT) session on the cognitive performance of amateur and elite players. Eighteen amateur and ten elite male basketball players were included in this study. They were asked to perform an acute SIT consisting of the Wingate Test (i.e., four bouts of 30 s all-out sprints) on a cycle ergometer, interspersed with 4 min of active recovery. Before and after the acute SIT, three cognitive tests (i.e., Change Detection Test, Timewall Test, Mackworth Clock Test) were performed. Exercise-induced changes in cognitive performance and between-group differences were analyzed. We did not observe significant between-group differences in the performance of any cognitive test at the pretest, but elite basketball players outperformed the amateur players in specific measures of the Change Detection Test and Timewall Test after the acute SIT (*p* < 0.05). In addition, for the Clock Test, only the elite basketball players’ performance improved from pre- to posttest. The current study’s findings suggest that male elite basketball players, compared to amateur basketball players, can preserve their cognitive performance after an acute bout of SIT.

## 1. Introduction

There is growing evidence suggesting that athletes not only require extraordinary physical abilities but also rely on superior cognitive abilities to compete at high levels of performance [1]. Indeed, the empirical evidence demonstrates that elite athletes outperform amateur athletes with respect to some specific cognitive abilities (e.g., executive functions) [1], especially those athletes that participate in a team-based sport [2]. In this context, there is also growing evidence that suggests that well-developed cognitive abilities are, among other factors, crucial for success in team sports such as soccer [3,4,5,6,7] or basketball [8,9,10]. For instance, Mangine et al. (2014) examined the relationship between basketball-specific performance parameters (e.g., assist, steal, turnover) and visual tracking speed (VTS) and reaction time (RT), according to different playing positions (e.g., forward or center), and observed that VTS was related to the performance level of many basketball-specific skills, such as passing or shooting [11]. Additionally, a previous study revealed a correlation between specific cognitive performance indices and shooting performance [10], while another study observed a link between decision-making performance and in-game performance [12]. Although the above-mentioned evidence strongly suggests that cognitive abilities are crucial in team sports such as basketball, the evidence concerning expertise-related differences (e.g., amateur athlete vs. elite athlete) is not exhaustive, as most of the available studies compared basketball athletes to non-athletes [13,14]. Thus, further research is necessary to reach a more comprehensive and nuanced understanding of the effect of the sport-specific expertise level on cognitive performance [1,15,16].

In this regard, there is solid evidence that an acute bout of physical exercise can improve cognitive performance in a variety of different cohorts [17,18], but our knowledge concerning possible mediators and moderators of this phenomenon is limited [17]. More specifically, there is also a gap in the literature on whether a single bout of exhaustive exercise (e.g., an acute bout of sprint interval training (SIT)) leading to a depletion of physical and cognitive resources might differently influence the cognitive performance of athletes of different expertise levels (i.e., amateur athletes vs. elite athletes).

Such a more comprehensive and nuanced understanding of how performance in distinct cognitive domains is influenced by the sport-specific expertise level and an acute bout of physical exercise would help to broaden our knowledge in the field of exercise-cognition in general and benefit athletic and sportive development as coaches, athletes, and sports researchers in particular as this knowledge can be applied in practice.

Among the several models that aim to explain the effects of physical exercises (e.g., SIT) on cognitive performance, the strength model of self-control proposed by Baumeister and colleagues is a popular option in exercise science [19,20]. The strength model of self-control postulates that there is a limited amount of self-control resources that can be depleted by tasks that require self-control resources (e.g., physical exercises) [20,21,22]. Moreover, the depletion of self-control resources, for instance, due to physical exercises (i.e., SIT), can worsen the performance in subsequent tasks that require self-control resources (i.e., specific cognitive tests being conducted after exercise cessation), [20,21,23]. However, the amount of self-control resources that are depleted by physical exercises depends on several factors such as the fitness level of the individual, exercise intensity, and exercise duration [20]. In general, a lower level of fitness, a higher exercise intensity, and a longer exercise duration are deemed to lead to an increased depletion of self-control resources (also known as ego-depletion) and, in turn, a more pronounced worsening in the performance of tasks that require self-control resources (i.e., specific cognitive tests being conducted after exercise cessation) [20]. With respect to the current study, the strength model of self-control would predict that amateur athletes who have a lower fitness level than elite athletes will show a higher depletion of self-control resources after a bout of strenuous physical exercise such as SIT and thus will perform worse than elite athletes in the subsequent cognitive tests (i.e., conducted after exercise cessation) [20].

In addition, we utilized SIT as an acute intervention as previous studies have shown that (i) SIT can improve cognitive performance (i.e., executive function) in younger adults [24], (ii) SIT can differently influence brain oxygenation patterns in athletes and controls [25], (iii) SIT mirrors to some extent the demands of the game (i.e., multiple field runs) of the cohort of athletes that is investigated in the current study (i.e., basketball athletes) and thus has frequently applied for testing purposes in this group of athletes [26,27,28], and (iv) SIT, due to all-out efforts, possibly consumes a large amount of self-control resources [20].

Taken together, this study aims (i) to investigate the potential differences between amateur and elite basketball players concerning their performance in specific cognitive tests and (ii) to study the effects of an acute bout of SIT on the cognitive performance of amateur and elite basketball players. Based on the available literature showing that elite athletes have, in general, superior cognitive performance abilities than amateur athletes [1], and in line with the strength model of self-control [20], we hypothesize that elite athletes outperform amateur athletes at rest (i.e., before an acute bout of exhaustive physical exercise) and after an acute bout of physical exercise.

## 2. Materials and Methods

### 2.1. Participants

Twenty-eight basketball players (18 amateurs, 20.38 ± 2.27 years; 10 elite, 20.80 ± 2.44 years) aged between 18 and 25 years participated in the current study. The elite basketball players in this study had at least 12 years of regular basketball training and competed for at least for 3 years on a professional level. Basketball players in the amateur group participated regularly in basketball for at least 8 years, but the amateur players had never played in any professional league. According to McKay et al. (2022), the elite basketball players recruited in this study can be classified as Tier 3, representing highly trained athletes who compete on a national level [29]. The amateur group can be classified as Tier 2, which include those who intend to compete in local-level competitions. No performance criteria or skill level is required to reach the latter classification [29]. None of the athletes did any specific cognitive training during their routine training sessions. In addition, all included athletes had no symptoms of neurologic, orthopedic, endocrinological, psychiatric, or cardiovascular disease and were not taking any medications.

The sample size in this study was determined by a combination of heuristic factors (i.e., orienting on the sample size of previous studies in the field such as [17,24,30] and resource constraints (i.e., the available number of elite athletes and time for doctoral research) rather than performing an a priori calculation of the sample size [31]. The sample size in the current study is constrained by (a) a limited number of available elite athletes, which is a common issue in studies investigating this cohort [1,32,33], (b) a time limit to complete the research for the doctoral thesis, and (c) the consequences arising from the measures against the spread of COVID-19 being present during the data collection (e.g., limited working hours and allowances, home confinement). In the current study, all measurements were taken between 10:30 and 12:00 A.M., and the participants were asked not to consume ergogenic foods and drinks (e.g., caffeine, alcohol, vitamin complexes) that could affect their cognitive performance nearly 2–3 h before the assessments. All subjects were informed about the procedures, and each gave written consent. Prior to the assessments, the experimental setup and the application of cognitive tests were explained to the participants.

### 2.2. Experimental Design

**Figure 1:**
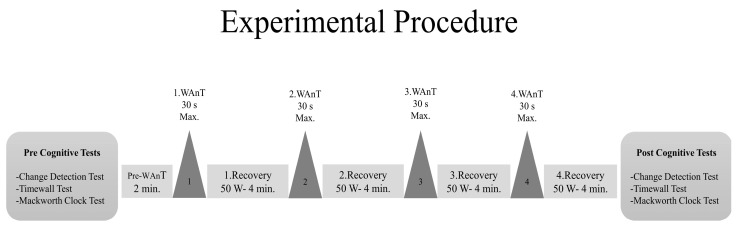
Experimental Procedure.

The current study was conducted in a between-subjects pretest-posttest comparison design [17]. First, the participant’s body composition (skeleton muscle mass, SMM; and body fat mass, BFM) was assessed using bioelectrical impedance measurements (InBody 720; Body Composition Analyzers, Seoul, Republic of Korea; [34]). These measurements were done to reveal the anthropometric differences between elite and amateur players. Afterward, three different cognitive tests were administered to the participants via a personal computer using the “Psychology Experimental Building Language” ((PEBL; [35,36]) test battery, and the results were recorded as the “pretest.” After the pretests, a SIT was performed (see Figure 1). The participants performed the same cognitive tests again 2 min. after the cessation of the last recovery bout of the SIT, and the results were recorded as the “posttest” (Figure 1). The heart rate (Polar RS 800, USA) and the perceived exertion (by using a Ratings of Perceived Exertion—RPE; [37]) were measured during the entire exercise session and recorded at the end of each minute. Additionally, at the end of each WAnT, HR, and RPE values were noted. Both the HR and RPE values obtained during the SIT were averaged.

### 2.3. Exercise Protocol

Following the completion of the pre-cognitive testing, a warm-up protocol was performed by the participants on a cycle ergometer, which included three short sprints in 5 min. Afterward, the participants were allowed to perform some self-selected stretching exercises. After the warm-up, the participants started pedaling for 2 min at 60–70 rpm at 50 W (the Pre-Want Session). The Wingate Anaerobic Test (WAnT), which is an all-out test to determine the level of anaerobic performance, was performed by all participants using a cycling ergometer (Monark 839E, Varberg, Sweden) four times with 4 min of active recovery (Rcy) between the repetitions [38,39]. During the WAnT, the participants were asked to use maximum effort, pedaling at a maximum speed for 30 s against a load of 80 g/kg of their body weight [40,41]. The entire exercise session lasted approximately 20 min (Figure 1). The Monark All-in-One software (ver.1.0.10.0) automatically calculated the peak power (PP) outputs of each participant, which were recorded during the WAnT [40].

### 2.4. Cognitive Tests

Three different cognitive tests were administered to the participants, before (pretest) and after (post-test) the cessation of the acute bout of physical exercises, as will be described in more detail in the following sections. Before the cognitive tests were administered, the participants were provided with a detailed explanation of the tasks and were informed about the procedures. To appropriately familiarize the participants with the cognitive tests and thus reduce learning-related effects, all participants completed a sufficient number of practice sessions [42]. Each cognitive test took about 3–5 min. The order of the tests was randomized (with respect to the different participants), but the order was kept constant for the pretest and posttest (with respect to the single participant). The participants were asked to sit at a distance of 70 cm from a 17-inch computer screen [43]. The cognitive test was performed in a quiet, well-lit room with only an empty wall, desk, PC screen, mouse, and keyboard in the participant’s visual field. The following monitor was used for cognitive testing: 17’’ W-LED Backlight LCD monitor with 1280 × 1024-pixel resolution, a refresh rate of 60 Hz, and a 5 ms response time (Philips, Inc., Amsterdam, Netherlands).

The applied cognitive tests were selected based on the following two criteria: (i) The selected cognitive test should assess the performance in cognitive domains that are related to basketball (i.e., timing ability) and might vary as a function of the expertise level. As currently there is not much evidence available on which cognitive domains are most important with respect to basketball performance [10,12], we selected the cognitive test by taking the opinions of expert basketball coaches and sports psychologists into account. (ii) To complement and add new evidence to the current literature, we used cognitive tests that are not that frequently applied in exercise-cognition research (for an overview of commonly used cognitive tests, see Pontifex et al. (2019) [17].

**Figure 2:**
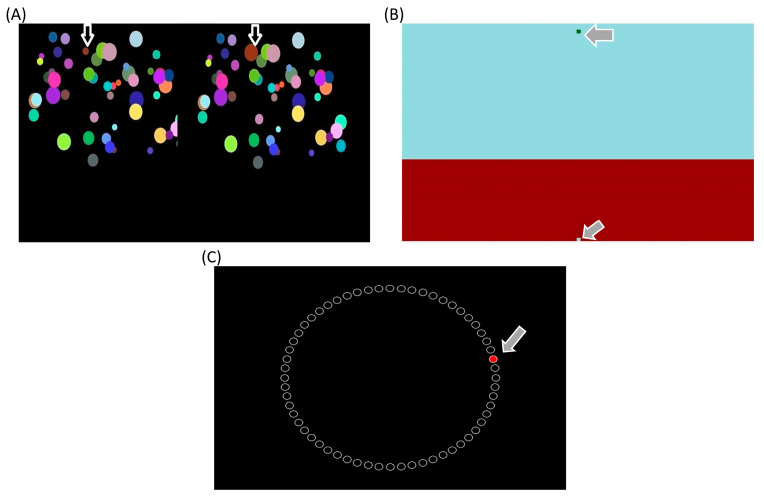
(**A**) Change Detection Test, (**B**) Timewall Test, and (**C**) Mackworth Clock Test.

The Change Detection Test measures visual spatial attention and working memory [35,44]. In this cognitive test, the participant was asked to recognize the change in two alternating visual scenes as soon as possible, which consisted of circles of various sizes and colors whose color, size, or position changed or disappeared in a flashing pattern on the screen (Figure 2A). The Change Detection Test consists of 20 trials. In this test, the response times of the participants and the number of correct answers were used to gauge cognitive performance (i.e., a faster response time and higher accuracy reflecting better performance). This test coincides with the necessity for basketball players to find the most accurate one among many changes in the game as soon as possible.

The Timewall Test is a nonverbal decision-making test that probes visual-spatial and time perception [35]. Moreover, the Timewall Test quantifies the timing skill performance, which is undoubtedly a critical skill in team sports (e.g., soccer, basketball) in general, and in basketball in particular [45,46]. This is a nonverbal decision-making test that probes visual-spatial perception and time perception [35]. The participant is expected to predict the time when a green rectangle with a width of 0.5 cm moving downwards with a constant velocity hits the ground behind a wall covering the lower third of the screen and to press the button when she/he thinks the green rectangle has hit the ground (Figure 2C). This cognitive test consists of 20 trials, and the performance of the participants was operationalized by an accuracy score, which is calculated via the ratio of the response time to the target time [Figure 2B; [47,48]]. A higher accuracy score in the Timewall Test reflects better decision-making performance.

The Mackworth Clock Test probes sustained attention and vigilance [35]. In this cognitive test, 60 small circles are ordered in a larger circular pattern (like a clock) on the test screen. A red pointer appears inside one of the small circles and jumps from one circle to the next (in a clockwise direction and in a one-by-one order). At infrequent and irregular time intervals, the red pointer skips a circle, and the participants were asked to respond as quickly as possible by pressing the space key when such an event occurs. The reaction time and the number of correct responses were used to quantify the cognitive performance in this test [Figure 2C; [49,50]], and a faster reaction time as well as higher accuracy reflect better performance.

### 2.5. Statistical Analysis

The JASP 0.16.2. (JASP Team, 2018; https://jasp-stats.org/, accessed on 31 August 2022) program was used for statistical analysis and to create the raincloud plots. The absence or presence of the normal distribution of the data was assessed using the Shapiro-Wilk test. According to the results of the Shapiro-Wilk test, some variables were normally distributed, whereas others were not. Therefore, we used non-parametric tests (i.e., the Wilcoxon test for within-group comparisons and the Mann Whitney-U test for between-group comparisons) for non-normally distributed data (i.e., performance indices of the Mackworth Clock test, experience [i.e., years of playing basketball], and demographic data). For the normally distributed data (i.e., skeletal muscle mass (SMM) and physiological variables and other cognitive test variables), parametric tests (i.e., paired sample t-tests for within-group analyses and independent samples *t*-tests for between-group analyses) were used. Additionally, we used a repeated measures ANOVA for the statistical analysis of the participants’ peak power values. All results were expressed as means (M) and standard deviations (SD). The significance level was set for all statistical tests at α < 0.05, and the effect sizes were reported as partial η2 (for ANOVA) or calculated with Cohen’s d (for dependent or independent t-test comparisons). Cohen’s d was rated as follows (with 95% confidence intervals): small effect <0.2, medium effect ≥0.2 to ≤0.8, and large effect >0.8 [51]. As for the non-parametric comparisons, the effect size is given by the rank biserial correlation on JASP. The rank biserial correlations r are rated as follows: <0.1: very small effect; ≥0.1 to ≤0.29: small effect; ≥0.3 to ≤0.49: medium effect; and ≥0.5: large effect [52].

## 3. Results

### 3.1. Demographic Information, Body Composition, and Physiologic Parameters

**Figure 3:**
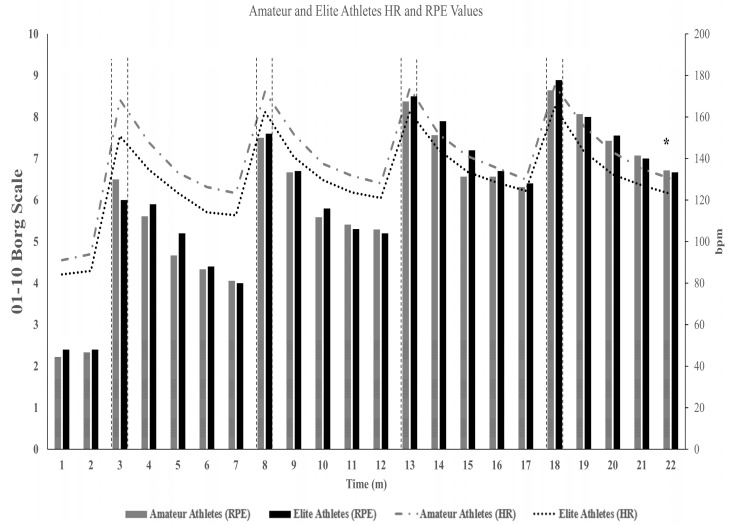
Mean of heart rate (HR) and ratings of perceived exertion (RPE) values of the amateur and elite basketball players during the entire SIT session. The left Y-axis shows the RPE (1–10 Borg Scale) values, while the right Y-axis shows HR values (in beats per minute). The X-axis shows the time in intervals of a minute, which starts with the beginning of the pre-want session and covers the whole SIT protocol. * denotes significant differences in HR values, *p* < 0.05.

**Figure 4:**
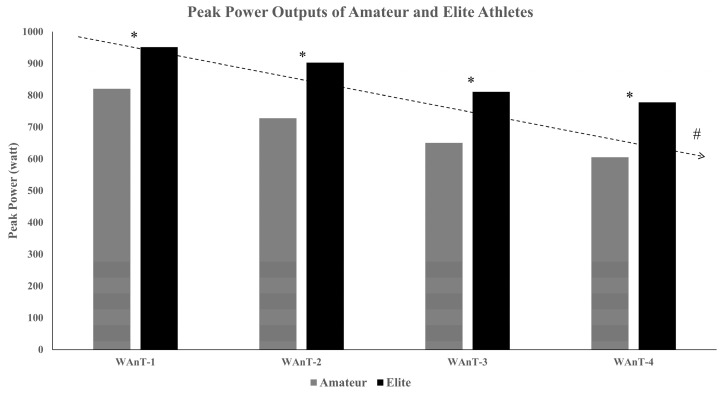
Peak power outputs of amateur and elite basketball players. * denotes significant differences between the amateur and elite groups, *p* < 0.05, # denotes a significant difference in the repetition within groups with a *p* < 0.05.

The general demographic and physiological characteristics of the participants are displayed in Table 1 and Figure 3 and Figure 4. For the sake of simplicity, the results section was divided into subsections to report the findings for physiological parameters and each of the three cognitive tests separately.

The analysis of the differences in basketball playing experience between the groups showed that the elite basketball players are significantly more experienced (U = 14, *p* < 0.001, r = −0.84) and have a more athletic body composition (SMM; t(26) = −2.57, *p* = 0.016, d = −1.01–BFM; U= 156, *p* = 0.002, r = 0.73) than the amateur players.

The heart rate of the elite basketball players during the entire exercise session was significantly lower than that of the amateur players [Figure 3; t(24) = −2.10, *p* = 0.046, d = 0.86]. There was no difference between the groups in terms of RPE values (t(26) = 0.87, *p* > 0.05, d = −0.34), (Figure 3). RPE and HR results show that amateur basketball players perform the same exercise with a higher heart rate but do not perceive a higher level of exertion. Despite the above-mentioned differences, no other statistically significant differences were observed concerning the RPE values.

### 3.2. Power Outputs

Concerning the peak power (PP) during the repeated Wingate test, a main effect of time was noticed, showing that the power outputs decrease with the increasing number of sprints [F(3.63) = 19.24, *p* < 0.001, η2 = 0.478, Figure 4]. Additionally, the PP output of the elite basketballers was significantly higher in each repetition as compared to the amateurs [F(1.21) = 8.96, *p* = 0.007, η2 = 0.299, Figure 4].

### 3.3. Cognitive Performance

#### 3.3.1. Change Detection Test Results

**Figure 5:**
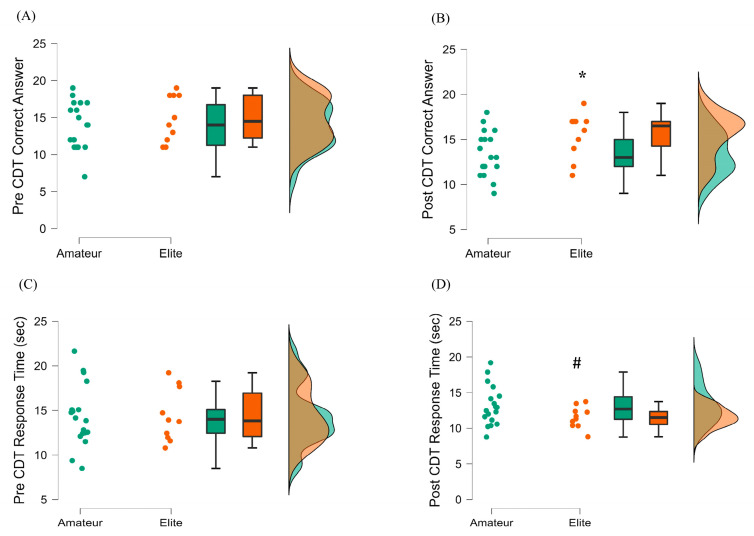
Change Detection Test (CDT) pre- and post-exercise comparisons between amateur (green) and elite basketball players (orange). (**A**) Pre CDT-Correct Answers (**B**) Post CDT-Correct Answers (**C**) Pre CDT-Response Time (**D**) Post CDT-Response Time * denotes significant differences between the amateur and elite groups, *p* < 0.05; # denotes within group pre-post comparisons with a *p* < 0.05.

At pretest, there were no statistically significant differences between the CDT correct answer scores and response times of amateur basketball players and elite basketball players. However, it was observed that the elite basketball players had a higher number of correct answers at the posttest as compared to amateurs [elite mean: 15.51 ± 2.51 and amateurs mean: 13.39 ± 2.50; t(26) = −2.13, *p* = 0.042, d = −0.84; Figure 5]. Although we observed significantly shorter response times in elite athletes at the posttest as compared to the pretest (t(9) = 3.31, *p* = 0.009, d = −1.04), there was no change in the percentage of correct answers (t(9) = 0.97, *p* > 0.05, d = −0.30). Concerning the amateur athletes, we noticed neither a statistically significant change in the percentage of correct answers nor a statistically significant change in response times when the pretest was contrasted with the posttest (t(17) = 1.76, *p* > 0.05, d = −0.41).

#### 3.3.2. Timewall Test Results

**Figure 6:**
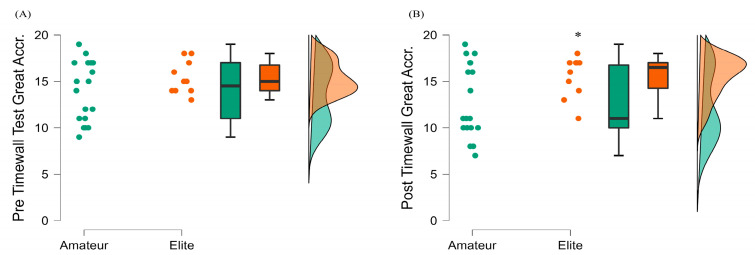
Timewall Test pre- and post-exercise comparisons between amateur (green) and elite (orange) basketball players. (**A**) Pre Timewall-Test Great Accuracy Scores (**B**) Post Timewall-Test Great Accuracy Scores * denotes significant differences after exercise between the amateur and elite groups, *p* < 0.05.

At pretest, no statistically significant differences were observed between amateur basketball players and elite basketball players concerning the accuracy score of the Timewall Test. However, elite basketball players achieved significantly better results at posttest as compared to the amateurs (elite mean = 15.50 ± 2.22 and amateurs mean = 12.83 ± 3.98; t(26) = 2.27, *p* = 0.032, d = −0.76; Figure 6). In addition, there was no statistically significant difference between the pretest and posttest timing scores of amateur and elite basketball players (t(17) = 1.91, *p* > 0.05, d = 0.45; t(9) = 0.12, *p* > 0.05, d = 0.040, respectively).

#### 3.3.3. Mackworth Clock Test Results

**Figure 7:**
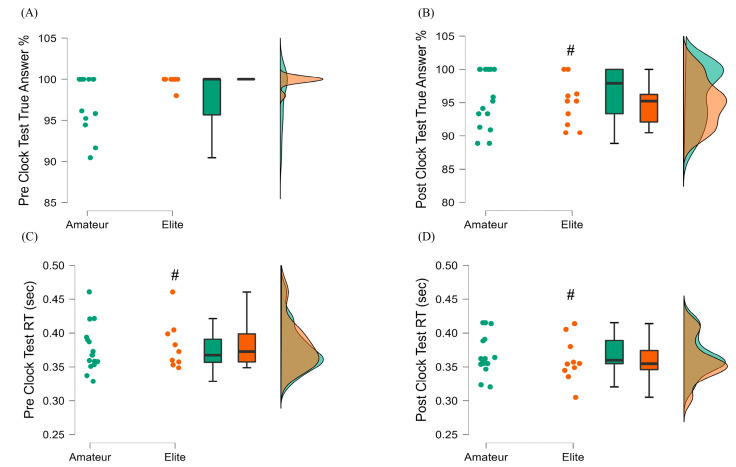
Mackworth Clock Test pre- and post-exercise comparisons between amateur (green) and elite (orange) basketball players. (**A**) Pre-Clock Test True Answers % (**B**) Post-Clock Test True Answers % (**C**) Pre-Clock Test Reaction Time (**D**) Post-Clock Reaction Time # denotes statistically significant different within group pre-post comparisons, *p* < 0.05.

Concerning the Mackworth Clock Test, no statistically significant between-group effects were registered (*p* > 0.05; Figure 7). Elite basketball players had shorter RTs at posttest as compared to pretest (W = 48, *p* = 0.037, r = −0.74). However, we also noticed a significant decrease in the percentage of correct answers in this group (W = 36, *p* = 0.012, r = 1.00). In addition, we did not observe a significant exercise-induced change in the performance indices of the Mackworth Clock Test of amateur basketball players (correct answers: W = 62, *p* > 0.05, r = 0.363; RT: W = 87, *p* > 0.05, r = −0.018; Figure 7).

## 4. Discussion

In this study, we examined (i) potential differences between male amateur and elite basketball players concerning their performance in specific cognitive tests and (ii) the effects of an acute bout of SIT on distinct measures of cognitive performance in these populations.

According to the results, elite basketball players had more muscle mass and less fat mass than amateurs, which is in line with the findings of previous studies [53,54]. Additionally, we found that elite basketball players had a higher peak power output (Table 1 and Figure 3) with a lower average heart rate (*p* < 0.05) and a comparable RPE than amateur basketball players (*p* > 0.05). These findings underpin the general observation that elite athletes (i.e., elite basketball players) have a superior physical fitness level [55,56] and they are able to achieve better performance (i.e., peak power output) with a lower internal load (i.e., a lower average heart rate and comparable RPE than amateur basketball players).

### 4.1. Cognitive Performance of Basketball Players

In the last decade, the physiologic and anthropometric developments of basketball players have been frequently investigated [57,58,59], while a limited number of studies focused on the influence of basketball on cognitive performance [1,11,15]. In the present study, we aimed to investigate (i) potential differences between male amateur and elite basketball players concerning their performance in specific cognitive tests and (ii) the effects of an acute bout of SIT on distinct measures of cognitive performance in these populations. With respect to the pretest, we did not observe a significant difference between the male amateur and elite basketball players, which is in slight contrast to the literature in which expertise-related differences concerning cognitive performance have been reported [1,2], although such an effect is rather of small-to-medium magnitude [1]. Accordingly, our finding might be related to the fact that the difference in the performance level (i.e., tier 3 vs. tier 2 according to McKay et al.’s (2022) classification framework) is too small so that significant cognitive performance differences do not occur in a resting state (i.e., pretest) in which cognitive resources are fully available [29]. Our finding adds to the rather mixed evidence on the effects of basketball on cognitive performance since the available cross-sectional studies report (i) that sub-elite male basketball players did not outperform non-athletes in a visuospatial attention task (i.e., the Corsi Block-tapping Task; [14]), whereas (ii) elite male basketball players have a superior performance in memory-retention, selective attention, and on prediction measures, but not speed of perception and response selection patterns, as compared to non-athletes [13].

However, when cognitive resources are depleted, for example, due to fatigue-related processes occurring in response to an acute SIT, expertise-related differences in measures of cognitive performance might be detectable. In line with the previous assumption, our findings show that male elite basketball players outperformed the amateurs at posttest (i.e., after the cessation of the acute SIT) with respect to the Change Detection Test and Timewall Test, which probed visual spatial attention and working memory as well as nonverbal decision-making, respectively. In addition, at the posttest, elite basketball players’ reaction times were faster in the Change Detection Test and Mackworth Clock Test as compared to the pretest, although the percentage of correct answers in the Mackworth Clock Test decreased from the pretest to the posttest. No such exercise-related changes were observed in amateur basketball players. The above-mentioned findings suggest that there are expertise-related differences in male basketball players concerning the performance in specific cognitive tests after a bout of exhaustive physical exercises (i.e., SIT) and thus, at least partly, support the idea to pay more attention to cognitive demands in the sport context (e.g., training monitoring; [60]). In this context, our finding that amateur basketball athletes did not show an improvement in cognitive performance is in slight contrast to a previous study demonstrating that acute SIT can improve cognitive performance in healthy younger adults [24]. However, these differences could be related to the fact that different cognitive domains (executive functions in [24] and visuospatial attention and decision-making in the current work) were studied and might be differentially influenced by acute physical exercises [17,24].

The between-group difference in cognitive outcomes at the posttest might be related to alterations on multiple levels of analysis, including changes on molecular and cellular levels (e.g., in the concentration of lactate, which has been associated with improvements in attentional performance after an acute bout of SIT in healthy younger individuals [30]) and/or changes on a functional brain level (e.g., in the prefrontal cortex). With respect to the latter, concerning elite athletes, Manci et al. (2021) showed that the cortical hemodynamics of athletes are different from those of sedentary individuals [25]. The latter finding is perhaps related to the assumptions outlined in the Neural Efficiency Hypotheses, which postulate that elite athletes are characterized by more efficient brain processes with respect to specific tasks [25]. According to the strength model of self-control [19,20,21,22], another potential explanation for our observations could be that, due to the lower fitness level of amateur athletes, a higher amount of self-control resources was depleted by the SIT, reducing the self-control resources available at the posttest, which might lead to the lower cognitive performance of amateur athletes as compared to elite athletes (i.e., at the posttest).

However, as the above-provided explanations of the process that might drive our behavioral findings are theoretical, further studies are needed to provide empirical evidence for those assumptions. In this study we did not assess changes on the molecular and cellular level nor on the functional brain level. Thus, we recommend that future studies aiming to replicate and extend our findings should use, for instance, functional neuroimaging techniques (e.g., functional near-infrared spectroscopy) to better understand the neural processes that drive change on the behavioral level and expertise-related differences.

### 4.2. Limitations

There are some limitations that need to be acknowledged. The cognitive tests that have been applied in this study were selected by taking the opinions of expert basketball coaches and sports psychologists. Due to this experience-based selection of cognitive tests, there might be some selection bias, and thus some of our cognitive tests might not be sensitive enough to detect expertise-related differences (e.g., the Mackworth Clock Test). However, as those cognitive tests are not frequently used in acute exercise-cognition studies [17], our findings complement and add new evidence to the literature. In addition, due to the time commitment needed to compete in the national leagues and the COVID-19-related conditions, it was difficult to reach a larger number of elite athletes, leading to the difference in the sample sizes of both groups. Thus, further studies should recruit a large sample size (if possible) to confirm, rebut, and/or extend our findings. Another limitation is the absence of a control condition (e.g., seated rest) for both groups. While the absence of the control condition somewhat hampers the conclusions that can be drawn with respect to possible time effects (i.e., learning effects), it is unlikely to influence or account for the observed expertise-related differences between elite and amateur athletes. In addition, as our sample and other samples [13,14] consist only of male basketball players, further studies should investigate whether our findings are generalizable to female basketball players.

## 5. Conclusions

The present study yielded two main findings: (i) there are no between-group differences concerning the performance in the Change Detection Test, Timewall Test, and Mackworth Clock Test at the pretest, and (ii) elite male basketball players outperformed amateur athletes after an acute bout of SIT with respect to the Change Detection Test and Timewall Test. In summary, our findings suggest that elite male basketball players, as compared to amateur basketball players, are able to preserve their cognitive performance in tests probing visuospatial attention and decision-making even after an exhaustive bout of acute physical exercises. Whether our findings are related to the superior neural efficiency and/or recovery ability of elite athletes needs to be determined in further studies.

## Figures and Tables

**Table 1:** Overview of demographic and physiological characteristics.

		N	Mean	SD. ±	t/U	Sig. (*p*)
Age	Amateur	18	20.3	2.2	-	-
	Elite	10	20.8	2.4		
Height (cm)	Amateur	18	187.0	10.2	−1.6	0.116
	Elite	10	192.8	5.5		
Weight (kg)	Amateur	18	87.7	16.1	−0.2	0.797
	Elite	10	89.2	10.3		
Years of Playing Basketball	Amateur	18	8.3	1.8	U = 14	<0.001 **
	Elite	10	11.8	1.8		
Professional Playing Year	Amateur	18	-	-	-	-
	Elite	10	3.2	1.9		
SMM (kg)	Amateur	18	40.9	5.9	−2.5	0.016 *
	Elite	10	47.0	6.0		
BFM (kg)	Amateur	18	15.6	8.6	U = 156	0.002 *
	Elite	10	3.2	1.9		
Mean HR (in bpm)	Amateur	18	140.3	13.4	2.1	0.046 *
	Elite	10	130.1	7.5		
Mean RPE	Amateur	18	5.9	0.7	−0.8	0.391
	Elite	10	6.1	0.5		

SMM: skeleton muscle mass, BFM: body fat mass, bpm: beats per minute, HR: heart rate, RPE: rating of perceived exertion (1–10), * *p* < 0.05, ** *p* < 0.01.

## Data Availability

The datasets generated during and/or analyzed during the current study are available from the corresponding author on reasonable request.
